# Variable Stiffness Fibers Enabled Universal and Programmable Re‐Foldability Strategy for Modular Soft Robotics

**DOI:** 10.1002/advs.202307350

**Published:** 2023-12-28

**Authors:** Hengxuan Luan, Meng Wang, Qiang Zhang, Zhong You, Zhongdong Jiao

**Affiliations:** ^1^ College of Mechanical and Electronic Engineering Shandong University of Science and Technology Qingdao 266590 China; ^2^ Shandong University of Science and Technology Taian 271019 China; ^3^ Department of Engineering Science University of Oxford Parks Road Oxford OX1 3PJ UK; ^4^ State Key Laboratory of Fluid Power and Mechatronic Systems Zhejiang University Hangzhou 310058 China

**Keywords:** origami, re‐foldability, soft actuators, soft robots

## Abstract

Origami is a rich source of inspiration for creating soft actuators with complex deformations. However, implementing the re‐foldability of origami on soft actuators remains a significant challenge. Herein, a universal and programmable re‐foldability strategy is reported to integrate multiple origami patterns into a single soft origami actuator, thereby enabling multimode morphing capability. This strategy can selectively activate and deactivate origami creases through variable stiffness fibers. The utilization of these fibers enables the programmability of crease pattern quantity and types within a single actuator, which expands the morphing modes and deformation ranges without increasing their physical size and chamber number. The universality of this approach is demonstrated by developing a series of re‐foldable soft origami actuators. Moreover, these soft origami actuators are utilized to construct a bidirectional crawling robot and a multimode soft gripper capable of adapting to object size, grasping orientation, and placing orientation. This work represents a significant step forward in the design of multifunctional soft actuators and holds great potential for the advancement of agile and versatile soft robots.

## Graphical abstract

Inspired by the re‐foldability of origami, a universal and programmable re‐foldability strategy that can integrate multiple origami patterns into a single soft origami actuator is reported. This strategy leverages variable stiffness fibers to selectively activate and deactivate origami creases, thereby enabling a series of re‐foldable soft origami actuators and multifunctional soft robots.

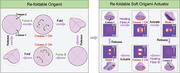

## 1 Introduction

Origami, the ancient art of paper folding, has served as a substantial source of inspiration for the development of compact and lightweight deployable systems. By incorporating flat rigid facets and well‐defined fold lines, origami can readily realize complex kinematic behavior and flat folding. These inherent advantages have led to its wide application across various fields such as DNA synthesis,^[^ ^1^, ^2^ ^]^ microfluidics,^[^ ^3^ ^]^ medical devices,^[^ ^4^, ^5^ ^]^ batteries,^[^ ^6^ ^]^ robotics,^[^ ^7^, ^8^, ^9^ ^]^ manufacturing,^[^ ^10^ ^]^ and space structures.^[^ ^11^, ^12^ ^]^ Recently, the integration of origami techniques into soft materials has simplified the design of soft actuators,^[^ ^13^, ^14^, ^15^, ^16^, ^17^ ^]^ endowing them with a variety of programmable functionalities, ranging from contraction, twisting, and bending, to radial morphing. However, these actuators are typically limited to a single origami morphing mode. By assembling multiple actuators into a soft machine and actuating them in different combinations, it is possible to achieve multimode morphing.^[^ ^14^, ^18^, ^19^, ^20^, ^21^, ^22^ ^]^ For example, the combination of an origami actuator capable of deformation coupling contraction and twisting enables the execution of contraction, twisting, and radial motions.^[^ ^14^ ^]^ Nevertheless, this approach often leads to a bulky structure. Therefore, there is a need to explore soft actuators with multimode morphing capability while maintaining a compact design.

In addition to foldability, origami also possesses re‐foldability, which allows the reversible transformation of a planar sheet into multiple 3D architectures. This characteristic provides a potential solution for designing compact soft actuators with multimode morphing capability. However, implementing re‐foldability on soft actuators necessitates the integration of multiple crease patterns within a single actuator and the selective activation of specific creases. The former can be realized by distributing multiple crease patterns into different surfaces to prevent mutual interference, while the latter can be achieved through the layer jamming of specific surfaces.^[^ ^23^ ^]^ Nevertheless, the layer jamming limits the morphing types of soft actuators, particularly in the case of complex origami morphing. For example, the twisting motion of the square‐twist origami cannot be achieved, because it requires the simultaneous activation of creases across multiple surfaces. Alternatively, varying the actuation sequences of two chambers can enable the bidirectional morphing of actuators with two creases on a single surface. However, the morphing modes are difficult to program and this method lacks generality.^[^ ^24^ ^]^ Consequently, a critical need arises for a universal strategy to design re‐foldable soft origami actuators with programmable morphing modes, thereby enriching the functionalities of soft machines.

In this work, we introduce a universal and programmable re‐foldability strategy to endow soft origami actuators with multimode morphing capability. This strategy allows multiple origami patterns to be integrated into one soft actuator, while selective activation of origami creases is realized via variable stiffness fibers. To validate the universality and programmability of this strategy, we build several soft origami actuators, including circular origami, rectangular frame origami, square‐twist origami actuators, and their combined configurations. The re‐foldability capability significantly enhances the deformation range of soft origami actuators without increasing their physical sizes and chamber numbers. Using these re‐foldable soft origami actuators, we create two multimode soft robots, including a bidirectional crawling robot and a multimode morphing soft gripper that can adapt to object size, grasping orientation, and placing orientation.

## 2 Results

### 2.1 Re‐Foldability Mechanism

**Figure 1:**
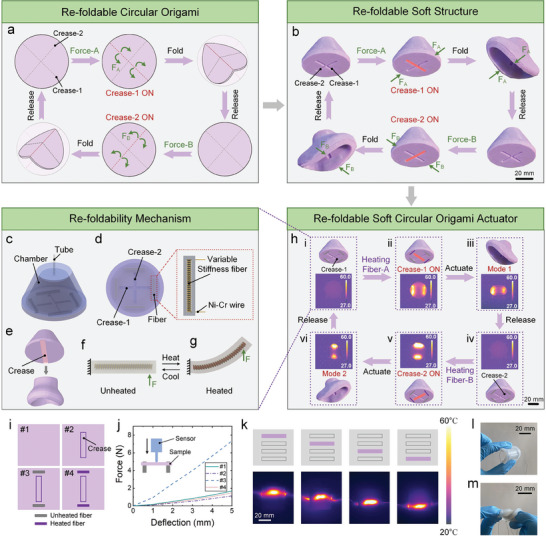
The mechanism of the re‐foldability is enabled by variable stiffness fibers. a) The re‐folding process of the circular origami. The red dotted line denotes the activated crease, and the gray dotted line denotes the deactivated crease. The selective activation/deactivation of creases is realized by exerting different folding forces on the paper. b) The re‐folding process of a soft structure inspired by circular origami. The selective activation/deactivation of creases is realized by applying pressing forces from different directions. c) The schematic illustration of the re‐foldable soft circular origami actuator. d) The bottom surface of the soft circular origami actuator. The inset shows the structure of the variable stiffness fiber. e) A soft origami actuator folds along its crease when subjected to vacuum pressure. f–g) The variable stiffness principle of the variable stiffness fiber. The fiber is in the rigid state before heating, while the fiber enters the soft state after heating. h) The re‐folding process of a soft pneumatic actuator. The selective activation and deactivation of creases are enabled by variable stiffness fibers. The insets are the infrared thermal images of the soft origami actuator. The numbers in these thermal images are temperature values. i) The four soft membrane samples with/without creases or fibers. #1: without creases and fibers; #2: with a crease only; #3 with a crease and unheated fibers; #4 with a crease and heated fibers. j) The force‐deflection curves of the four soft membrane samples. k) The infrared thermal images of a soft membrane with four fibers. The four fibers are heated sequentially. l–m) The soft membrane with creases and fibers can be bent and twisted.

**Figure** **1**a schematically illustrates the intrinsic re‐foldability characteristic of origami. A circular paper with two intersecting creases can be folded along different creases, thus exhibiting bidirectional contraction. This behavior is achieved by selectively applying different folding forces (Force‐A and Force‐B) to the circular paper. In addition, the two folding patterns can be reversibly transformed from each other.

The principle of re‐foldability can be applied to soft structures fabricated from soft materials (such as silicone rubber). As illustrated in Figure 1b, the soft structure inspired by the circular origami has two perpendicular grooves that function as creases. This structure is hollow inside and undergoes compression when subjected to pressing forces. Consequently, applying pressing forces from different directions will enable the bidirectional contraction morphing, which reproduces the re‐foldable motion of origami.

Soft materials possess the ability to actively morph when exposed to specific stimuli, such as fluidic pressure,^[^ ^14^ ^]^ light,^[^ ^25^, ^26^ ^]^ magnetic force,^[^ ^27^, ^28^, ^29^ ^]^ electricity,^[^ ^30^, ^31^, ^32^ ^]^ and chemical solvents.^[^ ^33^, ^34^, ^35^ ^]^ Therefore, it is possible to replace the pressing forces with these stimuli to facilitate the deformation of the aforementioned soft structure. In this work, we employ fluidic pressure as the stimulus to actuate the soft origami actuators. As shown in Figure 1e, a soft origami actuator folds along its crease when subjected to vacuum pressure. This behavior can be attributed to the fact that the vacuum pressure causes the actuator to collapse along the structurally weakest region. The introduction of the crease leads to a localized decrease in the stiffness, as evidenced by the mechanical characteristic comparison of sample #1 and #2 in Figure 1i‐j. However, it is noteworthy that when two crossed creases are incorporated into the actuator, it does not fold along either of the creases, as illustrated in Figure S11 (Supporting Information).

To select the desired creases for folding, we harness variable stiffness fiber^[^ ^36^, ^37^ ^]^ to selectively activate the required creases while deactivating the undesired ones. Specifically, the low melting point alloy (LMPA), a kind of phase‐change material, is selected as the variable stiffness fiber. The LMPA is a eutectic alloy composed of bismuth, indium, and tin. This alloy can be transitioned from a high‐stiffness solid state to a low‐stiffness liquid state at relatively low melting temperatures (the melting point is 60 °C). The subsequent cooling process can restore the fiber's stiffness to its original state. (Figure 1f‐g). As depicted in Figure 1i‐j, the sample with unheated fibers (sample #3) exhibits higher stiffness than that with heated fibers (sample #4). This observation suggests that the presence of unheated fibers effectively enhances the stiffness of the crease region. Moreover, the sample with heated fibers (sample #4) performs similar stiffness to that with only a crease (sample #2), demonstrating that the heated fibers have minimal influence on the mechanical properties of the soft membrane sample. These results imply that the variable stiffness fibers can activate and deactivate multiple creases by regulating the stiffness of crease regions.

As demonstrated in Figure 1c‐d, the variable stiffness fibers are fabricated by twining Ni–Cr resistance wire around a slender LMPA with a dimension of 2 mm × 2 mm × 20 mm. Subsequently, these fibers are embedded into the bottom surface of the soft origami actuator. Four variable stiffness fibers perpendicular to the creases are positioned at the crease terminations. An elastomer tube is used to regulate the internal pressure of the chamber. When the current is applied to the Ni–Cr resistance wire of Fiber‐A, this fiber is transitioned into the liquid phase with low stiffness and Crease‐1 is activated (Figure 1h‐ii). In contrast, Fiber‐B remains in its solid state, maintaining its high stiffness. Subsequent depressurization of the soft origami actuator leads to the folding motion along Crease‐1 due to the stiffness differences between Fiber‐A and Fiber‐B (Figure 1h‐iii; Movie S1, Supporting Information). Similarly, heating Fiber‐B activates Crease‐2 (Figure 1h‐v), leading to the folding motion along Crease‐2 upon applying the vacuum to the soft origami actuator (Figure 1h‐vi). Through the integration of variable stiffness fibers with vacuum‐induced buckling morphing, we successfully impart the re‐foldability characteristic of origami to soft actuators.

Furthermore, multiple variable stiffness fibers can be embedded into a single piece of soft membrane owing to the compact size of these fibers, as illustrated in Figure 1k. The elastomer between these fibers isolates them when heating and eliminates the mutual influences between them. Additionally, the soft membrane with creases and fibers can also be bent and twisted (Figure 1l,m).

### 2.2 Universality of the Re‐Foldability Strategy

**Figure 2:**
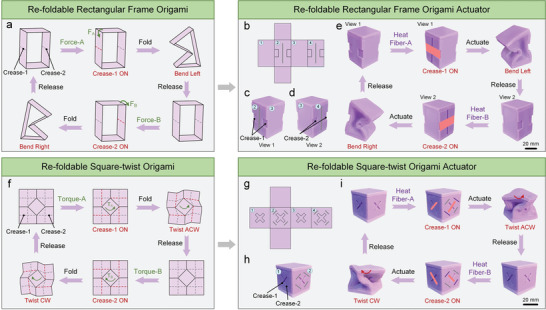
The universality of the re‐foldability strategy. a) The re‐folding process of the rectangular frame origami. The red dotted line denotes the activated crease, and the gray dotted line denotes the deactivated crease. b) The expanded view of the soft pneumatic actuator inspired by the rectangular frame origami. The creases and fibers are deployed on surface‐2 and surface‐4. c,d) Different views of the soft rectangular frame origami actuator. e) The re‐folding process of the soft rectangular frame origami actuator. The red area represents the activated crease. The purple line represents the heated fiber, and the gray line represents the unheated fiber. f) The re‐folding process of the square‐twist origami. g) The expanded view of the soft pneumatic actuator inspired by the square‐twist origami. h) The schematic diagram of the soft square‐twist origami actuator. i) The re‐folding process of the soft square‐twist origami actuator. The red arrow denotes the twisting direction of the actuator. CW and ACW are the abbreviations of clockwise and anticlockwise.

The re‐foldability strategy enabled by variable stiffness fibers can be applied to various soft origami actuators. As depicted in **Figure** **2**a, a rectangular frame origami with two creases deployed on opposite surfaces can bend left or right by folding along Crease‐1 or Crease‐2, respectively. Similarly, this origami can be converted into a pneumatic origami actuator by encapsulating the frame in an airtight chamber (Figure 2b–d). The two grooves on the left and right sides (surface‐2 and surface‐4) act as creases. Two variable stiffness fibers perpendicular to the creases are employed to activate/deactivate the two creases. The reversible and bidirectional bending deformation is demonstrated in Figure 2e; Figure S6a and Movie S2 (Supporting Information). The heating of Fiber‐A activates Crease‐1, leading to subsequent leftward bending morphing when subjected to vacuum pressure. Whereas the heating of Fiber‐B activates Crease‐2 and subsequent vacuum actuation leads to rightward bending morphing.

Circular origami and rectangular frame origami only require a single activated crease to perform bidirectional morphing. Furthermore, the compact size of the variable stiffness fibers allows the distribution and control of multiple creases in a single actuator to achieve more complex motion. An example is demonstrated by the square‐twist origami, which depends on four creases to realize twisting movements. As shown in Figure 2f, Crease‐1 (four anticlockwise creases) and Crease‐2 (four clockwise creases) are able to induce the anticlockwise and clockwise twisting of the origami upon folding, respectively. This origami can be converted into a soft square‐twist origami actuator with a cubic structure. Each side of the actuator has two intersecting creases, whereas only two opposite surfaces have four variable stiffness fibers to control the state of the creases (Figure 2g,h). Similarly, heating Fiber‐A activates the four anticlockwise creases, thus causing the soft origami actuator to rotate anticlockwise when subjected to vacuum pressure while heating Fiber‐B activates the four clockwise creases, which results in the clockwise rotation of the soft origami actuator upon evacuation (Figure 2i; Figure S6b, and Movie S3, Supporting Information).

### 2.3 Programmability of the Re‐Foldability Strategy

**Figure 3:**
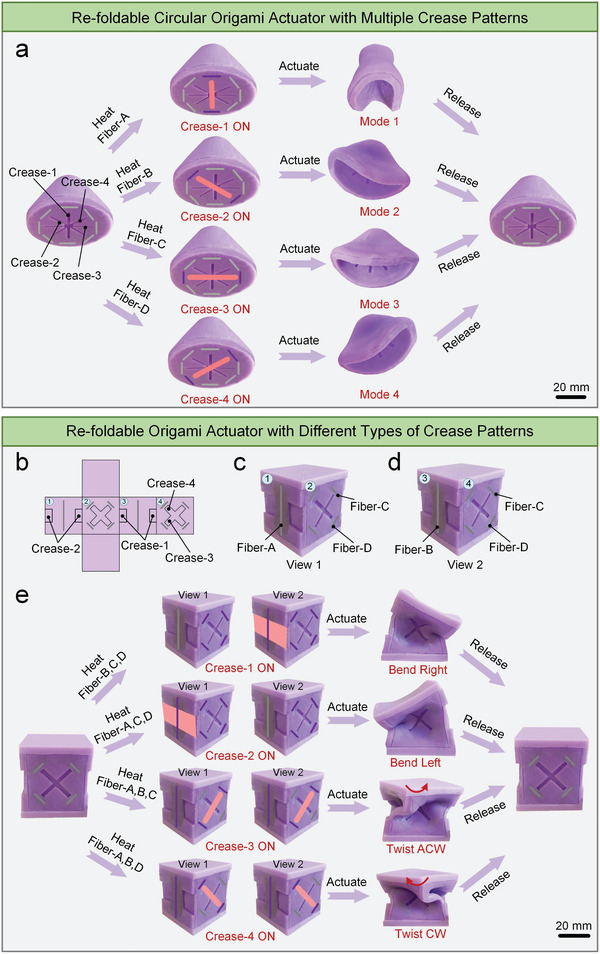
The programmability of the re‐foldability strategy. a) A re‐foldable circular origami actuator with multiple crease patterns. By activating and deactivating different creases, this actuator is able to exhibit four morphing modes reversibly. b) The expanded view of a cubic origami actuator with two rectangular frame origami patterns and two square‐twist origami patterns. c,d) Different views of the soft cubic origami actuator. e) The soft cubic actuator is capable of performing rightward bending, leftward bending, anticlockwise twisting, and clockwise twisting reversibly. The red area denotes the activated crease, the gray line denotes the unheated fiber, and the purple line denotes the heated fiber. CW and ACW are the abbreviations of clockwise and anticlockwise.

In addition, the compact size of the variable stiffness fibers endows this strategy with programmability in terms of crease pattern quantity and type. In **Figure** **3**a, four intersecting creases and four groups of variable stiffness fibers are deployed on the bottom surface of a circular origami actuator. Selective activation of these creases allows this actuator to reversibly switch between four morphing modes upon depressurization (Movie S4, Supporting Information).

As illustrated in Figure 3b–d, two rectangular frame origami patterns, and two square‐twist origami patterns are introduced into a soft cubic actuator. When Fiber‐B, Fiber‐C, and Fiber‐D are heated, only Crease‐1 is activated. Subsequent vacuum actuation causes this actuator to bend right (Figure 3e). Likewise, the heating of Fiber‐A, Fiber‐C, and Fiber‐D activates Crease‐2, resulting in the subsequent leftward bending. The heating of Fiber‐A, Fiber‐B, and Fiber‐C activates Crease‐3, leading to anticlockwise twisting. Lastly, the heating of Fiber‐A, Fiber‐B, and Fiber‐D activates Crease‐4, causing clockwise twisting when the actuator is vacuumed (Movie S5, Supporting Information).

The programmability in crease pattern quantity and type could potentially unlock a vast array of soft origami actuators with versatile multimode morphing capabilities. Besides, these soft origami actuators can retain their multimode morphing functionality even when they are scaled up/down in dimensions, as displayed in Movies S6 (Supporting Information).

### 2.4 Stiffness and Actuation Characteristics

**Figure 4:**
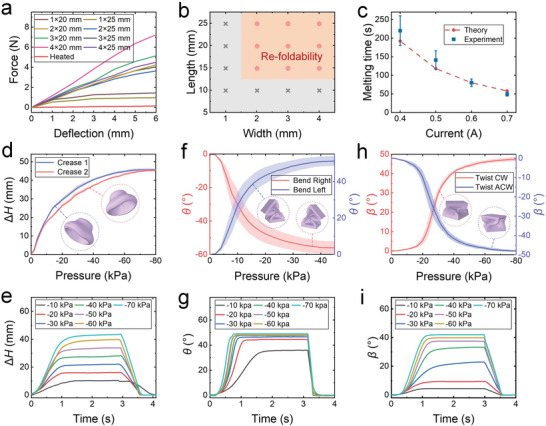
The stiffness and actuation characteristics of the re‐foldable soft origami actuators. a) The force‐deflection curves of the unheated variable stiffness fibers with different widths and lengths. The cross‐sections of these fibers are squares. b) The influence of fiber width and length on the re‐foldability of soft actuators. The red circle means that the actuator can perform re‐foldable morphing, and the gray cross means that the actuator fails to perform re‐foldable morphing. c) Melting time of the variable stiffness fibers that are heated by different constant currents. The experimental results are compared with the theoretical values calculated by Equation (2). d) The contraction stroke *△H* of the soft circular origami actuator as a function of the actuation pressure. e) The effect of the actuation pressure on the deformation response of the soft circular origami actuator. f) The bending angle *θ* of the soft rectangular frame origami actuator as a function of the actuation pressure. g) The effect of the actuation pressure on the deformation response of the soft rectangular frame origami actuator. h) The twisting angle *β* of the soft square‐twist origami actuator as a function of the actuation pressure. i) The effect of the actuation pressure on the deformation response of the soft square‐twist origami actuator.

Since the variable stiffness fibers play crucial roles in re‐foldable morphing, we investigated the dependence of bending stiffness on fiber width. According to cantilever beam equations, the bending stiffness *EI* of the variable stiffness fiber can be expressed as:

(1) $$EI=\frac{FL^{3}}{3\delta_{b}}=\frac{L^{3}}{3}\frac{F}{\delta_{b}}$$

where *F* represents the force applied on the fiber, *L* denotes the length of the fiber, *δ_b_* is the deflection of the fiber, *E* is the equivalent elastic modulus, and *I* is the moment of inertia. The bending stiffness of the fiber can be determined by the slope of the force‐deflection curves. It is apparent from **Figure** **4**a that the bending stiffness of the fiber increases as the width increases. Besides, the bending stiffness of heated and unheated fibers with a size of 3 mm × 25 mm was also compared, where the unheated fiber performed ≈29 times the bending stiffness of the heated fiber.

The soft actuators presented in this work depend on the stiffness variation of the fibers to realize the re‐foldable morphing. Consequently, it is crucial to carefully select the size of the fibers to meet the desired requirements. We conducted experiments to investigate the dependence of re‐foldability on fiber length and width. As demonstrated in Figure 4b, both the length and width have a significant impact on the deformation of the actuators. When the fiber length reaches 15 mm and the fiber width reaches 2 mm, the soft actuators are able to exhibit re‐foldable morphing.

The response speed of stiffness variation in the fibers is determined by the heating current. The melting time of the fiber can be estimated by balancing the power required to melt the alloy, the electric power supplied to the external heater, and the power lost due to convection in the surrounding air (according to Newton's law of cooling):

(2) $$t=\frac{\left(m_{ela}c_{ela}+m_{LMPA}c_{LMPA}\right)\Delta T+m_{LMPA}L_{LMPA}}{\eta {I_{heat}}^{2}R-hA\Delta T}$$

where m_LMPA_, c_LMPA_, and *L*_LMPA_ represent the mass, specific heat, and latent heat of fusion of the LMPA, respectively. m_ela_ and c_ela_ are the mass and specific heat of the elastomer. ΔT is the temperature difference between the melting temperature of the fiber and room temperature. h and A denote the heat transfer coefficient of air and heat transfer surface area, respectively. I_heat_ is the heating current of the heater, *ƞ* is the heating efficiency, and R is the resistance of the heater.

The resistance of the heater, made of Ni–Cr resistance wire, can be determined using the laws of resistance:

(3) $$R=\frac{\rho_{wire}l_{wire}}{S}=\frac{\rho_{wire}l_{wire}}{\pi r_{wire}^{2}}$$

where ρ_wire_ represents the resistivity of the Ni–Cr resistance wire, l_wire_ is the wire length, r_wire_ is the wire radius, and *S* is the cross‐sectional area of the wire. As depicted in Figure 4c, the melting time decreased with the increased heating current, and the calculated heating time agrees well with the experimental values.

The presented re‐foldability strategy efficiently increases the deformation capability of current single‐chamber actuators. ^[^ ^13^, ^16^, ^38^ ^]^ As depicted in Figure 4d, the soft circular origami is able to exhibit bidirectional contraction along the direction perpendicular to the activated creases. The maximum contractions in the two directions are 45.7 and 45.2 mm, respectively. It is apparent that the relationship between vacuum power and the contraction stroke △*H* of the actuator is nonlinear. The contraction stroke △*H* and response speed of the soft circular origami actuator increase with the increased vacuum pressure (Figure 4e).

The soft rectangular frame origami actuator can perform bidirectional bending morphing ranging from −56.0° to 51.2°. As shown in Figure 4f‐g, the bending angle *θ* and response speed initially show a significant increase when the actuation pressure is higher than −30 kPa. However, beyond this threshold, the bending angle *θ* and response speed show only a slight increase with further increased vacuum power.

In Figure 4h, the re‐foldability allows the soft square‐twist origami actuator to twist from −47.4° to 48.0°. The twisting angle *β* increases nonlinearly with the decrease of the actuation pressure in both twisting directions. As depicted in Figure 4i, higher vacuum power leads to faster response speed. When powered by an actuation pressure of −60 kPa, the actuator reaches its deformed state (90% of the maximum twisting angle) within ≈1.01 s, while in the restoring process, it takes ≈0.51 s to return to its original state (10% of the maximum twisting angle).

Besides, continuous operation for 1000 cycles under a frequency of 0.25 Hz was carried out to test the stability of these soft origami actuators. As depicted in Figure S5 (Supporting Information), the contraction, bending, and twisting deformations remained stable during the test and no mechanical failure or permanent changes were observed, demonstrating their excellent repeatability and long service life.

The output force and torque are important performance characterizations for soft actuators. We measured the contraction force, bending force, and torque generated by these re‐foldable soft origami actuators. As depicted in Figure S10 (Supporting Information), the contraction force, bending force, and torque exhibit an increasing trend with higher vacuum pressure.

The creases make the soft actuators fold along them when powered by vacuum pressure. Then the effect of crease width, length, depth, and density on the deformation of the actuators is investigated by finite element analysis. The results show that these parameters have few influences on the maximum deformation of the actuators, as shown in Figure S12 (Supporting Information). Therefore, the creases integrated within the actuators only serve as triggers to induce the desired collapse states. When subjected to vacuum pressure, the actuators collapse along the activated creases.

### 2.5 Multimode Locomotion Enabled by Re‐Foldability Strategy

#### 2.5.1 Bidirectional Crawling Robot

**Figure 5:**
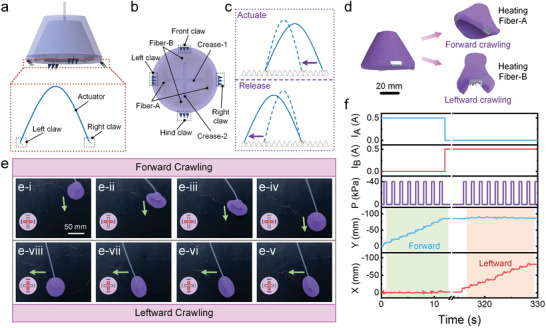
A bidirectional crawling robot constructed with a soft circular origami actuator. a) The schematic illustration of the soft crawling robot. Four claws are evenly deployed around the circumference. The inset is the simplified view of the crawling robot. b) The bottom structure of the crawling robot (upward view). The two claws on opposite sides bend in the same direction. c) The operational principle of the crawling robot. d) The deformation of the crawling robot in the forward and leftward crawling gaits. e) The bidirectional movement of the crawling robot. The states of the fibers and creases are shown in the bottom‐left corner. f) The trajectory in the *X* and *Y* directions of the crawling robots. *I*_A_ and *I*_B_ denote the currents that power Fiber‐A and Fiber‐B. *P* denotes the actuation pressure of the crawling robot.

The re‐foldability strategy empowers the soft origami actuators to exhibit various multimode deformations, which can be further utilized to create a variety of soft robots capable of multimode locomotion. As schematically illustrated in **Figure** **5**a, a soft crawling robot is constructed by attaching four inclined claws to the circular origami actuator. In this design, the two claws on opposite sides bend in the same direction, resulting in different friction forces in opposite directions (Figure 5a,b).

When Fiber‐B is heated, Crease‐2 enters the activated state (Figure 5b). Subsequently, the evacuation of the chamber causes the circular origami actuator to contract, storing the elastic energy in the soft materials. This contraction causes the right claw to move left due to the anisotropic friction. In the subsequent releasing process, the stored elastic energy is released and the actuator stretches, propelling the left claw to move left due to the larger friction at the right claw. The actuation and releasing processes form a locomotion cycle, enabling the crawling robot to move leftward (Figure 5c,d). Upon heating Fiber‐A, Crease‐1 is activated and the same actuation and releasing processes lead to another locomotion mode: forward crawling (Figure 5d).

The continuous locomotion of moving forward and leftward is demonstrated in Figure 5e,f and Movie S7 (Supporting Information). This crawling robot can achieve a locomotion speed of 7.19 mms^−1^ (7.19 BL min^−1^) when powered by an actuation pressure of −40 kPa.

#### 2.5.2 Multimode Morphing Soft Gripper

**Figure 6:**
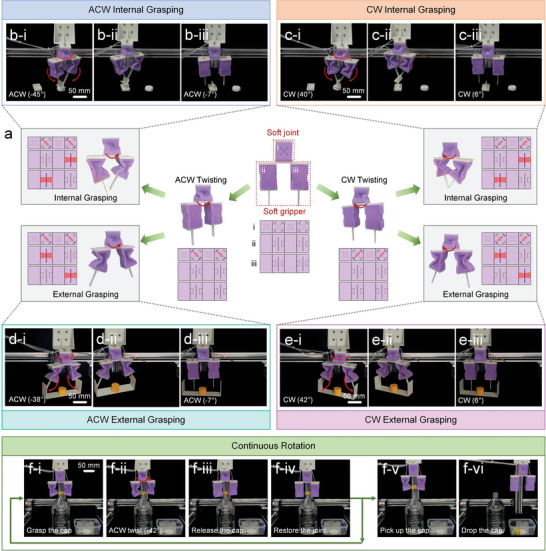
A soft gripper with multimode morphing capability. a) The soft gripper can perform clockwise and anticlockwise twisting, and internal and external grasping by selectively activating/deactivating specific creases. The fiber and crease states are depicted in the expanded views of the soft origami actuators. b,c) The soft gripper twisted anticlockwise and clockwise, picked up a small object through internal grasping, and placed the object into a rectangular hole. d,e) The soft gripper twisted anticlockwise and clockwise, picked up a large object through external grasping, and placed the object on the ground. f) The soft gripper unscrewed a cap by continuous anticlockwise twisting. CW and ACW are the abbreviations of clockwise and anticlockwise.

In the second application, a multimode morphing soft gripper is constructed by combining two soft rectangular frame origami actuators (**Figure** **6**a‐ii,iii) with a square‐twist origami actuator (Figure 6a‐i). In this design, the rectangular frame origami actuators are configured for grasping, while the square‐twist origami actuator functions as a soft joint. By selectively activating/deactivating creases via the variable stiffness fibers, this gripper can twist clockwise or anticlockwise and bend inward or outward, therefore adapting to various grasping tasks.

The inward bending mode enables the gripper to manipulate small objects (Figure 6b,c; Movie S8, Supporting Information), while the outward bending mode empowers it to grasp larger structures (Figure 6d,e; Movie S8, Supporting Information). The grasping force can be modulated by regulating the actuation pressure of the soft rectangular frame origami actuators, as depicted in Figure S9 (Supporting Information). The twisting capability allows the gripper to adjust the grasping and placing orientations by controlling the actuation pressure of the square‐twist origami actuator. As demonstrated in Figure 6b,c, the soft gripper grasped objects with different orientations and placed them into holes with specific shapes and orientations.

In addition, the combination of the bending and twisting deformations allows this gripper to perform complex tasks, such as unscrewing a bottle cap. In Figure 6f and Movie S9 (Supporting Information), the gripper exhibited continuous rotation by repeatedly executing four consecutive operations: grasping the cap, twisting anticlockwise, releasing the cap, and restoring the soft joint. After unscrewing the cap, the gripper picked up the cap and dropped it into a box.

## 3 Conclusion and Discussion

In this work, we present a universal and programmable re‐foldability strategy to confer soft origami actuators with the ability to exhibit multimode morphing. This innovative strategy allows multiple origami patterns to be integrated into a single soft actuator by introducing variable stiffness fibers that can selectively activate or deactivate specific creases. We developed three re‐foldable soft origami actuators: circular origami, rectangular frame origami, and square‐twist origami actuators, demonstrating the universal applicability of our approach. The variable stiffness fiber also allows the crease pattern quantity and types in a single actuator to be scaled up, thereby further expanding the morphing modes. Importantly, this re‐foldability technique effectively broadens the deformation range of soft origami actuators without increasing physical size and chamber number. Furthermore, we harness these re‐foldable soft origami actuators to construct a range of multimode soft robots, including a bidirectional crawling robot and a highly adaptable soft gripper capable of accommodating object size, grasping orientation, and placing orientation variations.

The re‐foldability strategy, activating/deactivating origami creases via variable stiffness fibers, is a universal approach to converting multimode origami into multimode soft origami actuators. Although Lin et al. employed layer jamming to enable multimode morphing, this method restricts each surface to possessing only one crease, because the entire surface undergoes hardening during the layer jamming process.^[^ ^23^ ^]^ In comparison, our variable stiffness fibers only require thin fluidic tunnels to locally control stiffness, making it possible to integrate multiple creases within a single surface and selectively activate these creases. It is worth mentioning that the presented re‐foldability strategy is not limited to fluidic actuation. Other actuation technologies, such as magnetic actuation,^[^ ^39^, ^40^, ^41^ ^]^ dielectric elastomer actuation,^[^ ^42^, ^43^ ^]^ electro‐hydrodynamic actuation,^[^ ^44^, ^45^, ^46^ ^]^ and hydraulic electrostatic actuation,^[^ ^47^, ^48^, ^49^ ^]^ can also be harnessed to achieve the multimode morphing demonstrated in this study.

The re‐foldability strategy allows the soft origami actuators to effectively double their deformation range without increasing size and chamber numbers. Moreover, this strategy exhibits programmability, as the morphing range and type can be further expanded by embedding more crease patterns and variable stiffness fibers.

The modular design of the presented soft origami actuators allows them to be readily combined into soft machines to achieve enhanced functionalities. As a prototype demonstration, we integrated the square‐twist origami and rectangular frame origami modules to construct soft grippers capable of adapting to complex grasping tasks, involving variations in sizes, shapes, grasping orientation, and placing orientations. We envision that these soft origami actuators have the potential to significantly enhance the agility and efficiency of current soft robots.

Despite the relatively slow response speed exhibited by the proposed variable stiffness fibers, recent advances in electrostatic jamming^[^ ^50^ ^]^ offer possibilities to realize high‐speed variable stiffness fibers for re‐foldable soft origami actuators. The output force and torque generated by these soft origami actuators are comparatively smaller than those produced by current pneumatic actuators. However, these performance characterizations can be further enhanced by fabricating these soft actuators using elastomers with higher elastic modulus.

## 4 Experimental Section

### Fabrication of the Variable Stiffness Fibers

The manufacturing process of the variable stiffness fibers is depicted in Figure S2a (Supporting Information). The alloy blocks (HiTech Alloys) with a melting temperature of 60 °C and a composition by weight of 51% In, 32.5% Bi, and 16.5% Sn were placed on a heated plate set at a temperature of 120 °C, causing the alloy to melt. The liquid alloy was then poured into a mold and became the desired fiber shape. The fiber was removed from the mold when cooled to the solid state. Subsequently, a Ni–Cr resistance wire (Menghe Nuoya New Material) with a radius of 0.05 mm and a resistivity of 138.8 Ω m^−1^ was wound around the fiber at a rate of 5 turns per centimeter. This Ni–Cr resistance wire was used for the Joule heating of the fiber.

### Fabrication of the Re‐Foldable Origami Actuators

The elastomer parts of the soft origami actuators were fabricated by elastomer casting, as demonstrated in Figure S2b–d (Supporting Information). The molds were made of polylactic acid (PLA) and printed in a customized fused deposition modeling (FDM) 3D printer. The casting process comprised three steps: 1) Two components of elastomers (E620, Shenzhen Hongyejie Technology Co., Ltd.) and pigment were poured into a beaker and mixed with a glass rod. The mixture was degassed in a vacuum container to remove the air in it. 2) The liquid elastomer mixtures were poured into the molds and cured at room temperature. 3) The cured elastomers were removed from the molds and joined together using silicone adhesive (441, Ausbond). The soft membranes with embedded variable stiffness fibers (Part‐2 in Figure S2b–d, Supporting Information) were fabricated via two casting processes.

### Thermal Behavior of the Variable Stiffness Fiber

An infrared thermal camera was used to record the temperature changes during the heating and cooling processes of the variable stiffness fibers. As shown in Figure S3 (Supporting Information), when a current of 0.6 A was applied to the Ni–Cr resistance wire, the local temperature increased to the melting point within 2 min. Subsequently, the variable fiber was completely liquefied, exhibiting a significantly reduced stiffness. The temperature continued to rise beyond the melting point. Upon ceasing the power supply to the heater, the temperature of the liquid fiber started to drop, transitioning back into the solid phase. At a room temperature of 30 °C, the phase transition from liquid to solid was completed within 2.5 min.

### The Stiffness Characteristic of the Variable Stiffness Fiber

The stiffness of the variable stiffness fibers was determined through cantilever beam experiments, as demonstrated in Figure S4 (Supporting Information). In this experimental setup, a cantilever beam (variable stiffness fiber) was fixed at one end and free at the other. By pressing the free end of the beam with a force gauge (ZNLBM‐IIX, Zhongnuochuanli), the force‐deflection of variable stiffness fibers was obtained.

### Finite Element Analysis

To accelerate the optimization design process of the re‐foldable soft origami actuators, finite element analysis (FEA) simulations were conducted using the Explicit solver within the commercial software package ABAQUS. In these simulations, all soft origami actuators were modeled as elastomeric chambers integrated with variable stiffness fibers. One end of the actuator was completely fixed, while the inner surface of the actuator was subjected to a pressure load to simulate the morphing behavior. Yeoh hyperelastic material and linear elastic material were assigned to the elastomeric parts and variable stiffness fibers, respectively. The actuators were meshed using 8‐node linear reduced‐integration hexahedral elements with a hybrid formulation (C3D8R).

### Control System for the Re‐Foldable Origami Actuators

The re‐foldable origami actuators and multimode morphing robots were regulated by a custom‐built control system. As illustrated in Figure S8 (Supporting Information), the actuation sequences of these soft machines were programmed in a computer and executed in a microcontroller (Arduino MEGA2560 R3). The control signals sent from the microcontroller were transmitted to the electrical relays, which actuate the three‐way solenoid valves (0520F, Weilizi) and the Ni–Cr resistance wires directly. The vacuum pressure was generated by a vacuum pump (JBL‐1100 W, Jiabaoli) and regulated via the pressure regulator (ITV 2090, SMC). The variable stiffness fibers were heated by a DC power supply via electrical relays (YYG‐2, SONGLE).

### Measuring Methods for the Re‐Foldable Origami Actuators

The temperatures of the actuators were recorded with an infrared thermal imaging camera (T1050sc 28, FLIR). The contraction stroke, bending angle, and twisting angle of the actuators were recorded using a camera. The deformation variables are depicted in Figure S7 (Supporting Information). The contraction force and bending force were measured using a force gauge (ZNLBM‐IIX, Zhongnuochuanli). The torque was measured with a torque tester (ST‐1, SHSIWI).

## Conflict of Interest

The authors declare no conflict of interest.

## Supporting information

Supporting Information

Supplemental Movie 1

Supplemental Movie 2

Supplemental Movie 3

Supplemental Movie 4

Supplemental Movie 5

Supplemental Movie 6

Supplemental Movie 7

Supplemental Movie 8

Supplemental Movie 9

## Data Availability

The data that support the findings of this study are available from the corresponding author upon reasonable request.
