# Supplementary material for: Variable Stiffness Fibers Enabled Universal and Programmable Re‐Foldability Strategy for Modular Soft Robotics
Source: Adv Sci (Weinh). 2023 Dec 28;11(10):2307350. doi: 10.1002/advs.202307350 (PMC10933646; doi:10.1002/advs.202307350)
Supplement: Supplementary file 1 — Supporting Information [file ADVS-11-2307350-s002.pdf]

ADVANCED SCIENCE
Open Access

Supporting Information

for *Adv. Sci.*, DOI 10.1002/advs.202307350

Variable Stiffness Fibers Enabled Universal and Programmable Re-Foldability Strategy for Modular Soft Robotics

*Hengxuan Luan, Meng Wang, Qiang Zhang, Zhong You and Zhongdong Jiao**

Supporting Information

Variable stiffness fibers enabled universal and programmable re-foldability strategy for modular soft robotics

Hengxuan Luan, Meng Wang, Qiang Zhang, Zhong You, Zhongdong Jiao*

*Email of Corresponding Author: zdjiao@zju.edu.cn

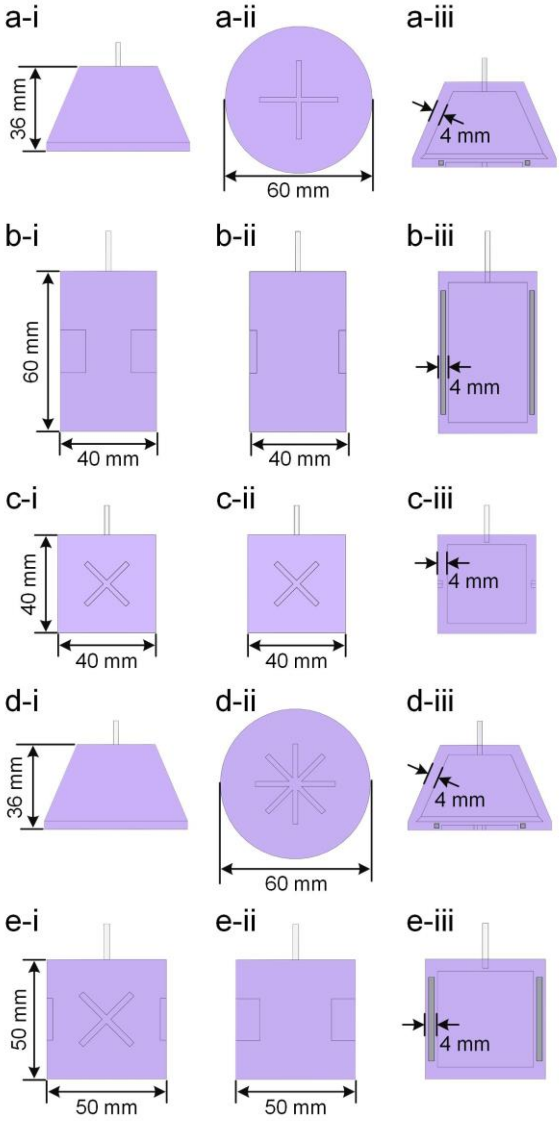

Figure S1. The dimensions and internal structure of the soft origami actuators. (a) The soft circular origami actuator. (a)-i The front view. (a)-ii The upward view. (a)-iii The cross-section view. (b) The soft rectangular frame origami actuator. (b)-i The front view. (b)-ii The side view. (b)-iii The cross-section view. (c) The soft square-twist origami actuator. (c)-i The front view. (c)-ii The side view. (c)-iii The cross-section view. (d) The soft circular origami actuator with multiple crease patterns. (d)-i The front view. (d)-ii The upward view. (d)-iii The cross-section view. (e) The soft cubic origami actuator with two rectangular frame origami patterns and two square-twist origami patterns. (e)-i The front view. (e)-ii The side view. (e)-iii The cross-section view.

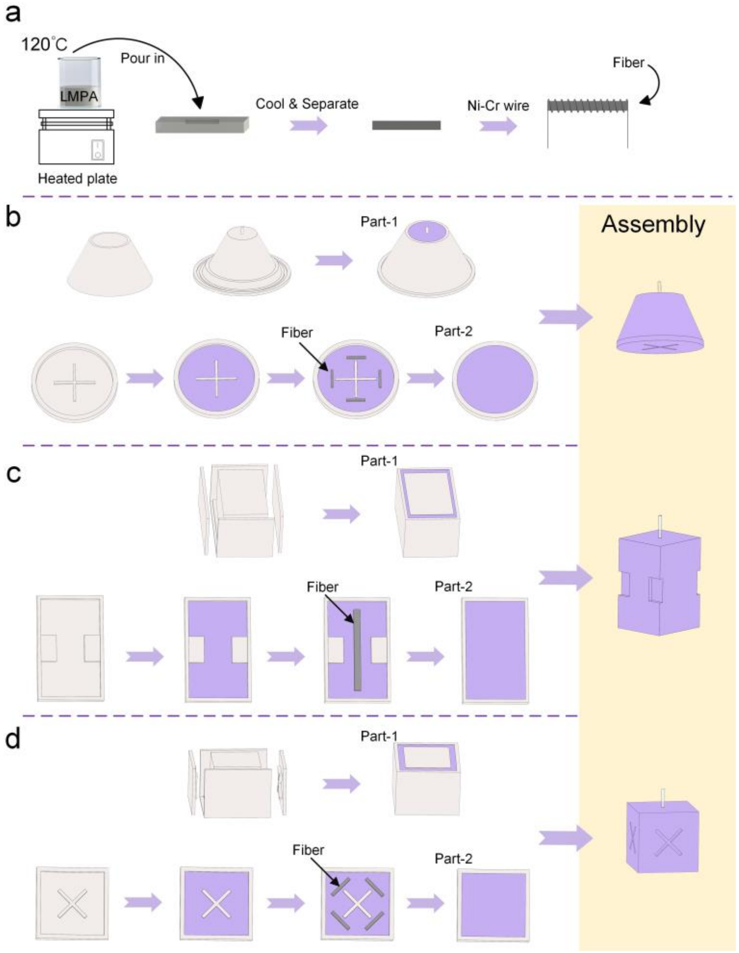

Figure S2. The fabrication process of the re-foldable origami actuators. (a) The fabrication of the variable stiffness fibers. (b) The fabrication of the soft circular origami actuator. (c) The fabrication of the soft rectangular frame origami actuator. (d) The fabrication of the soft square-twist origami actuator.

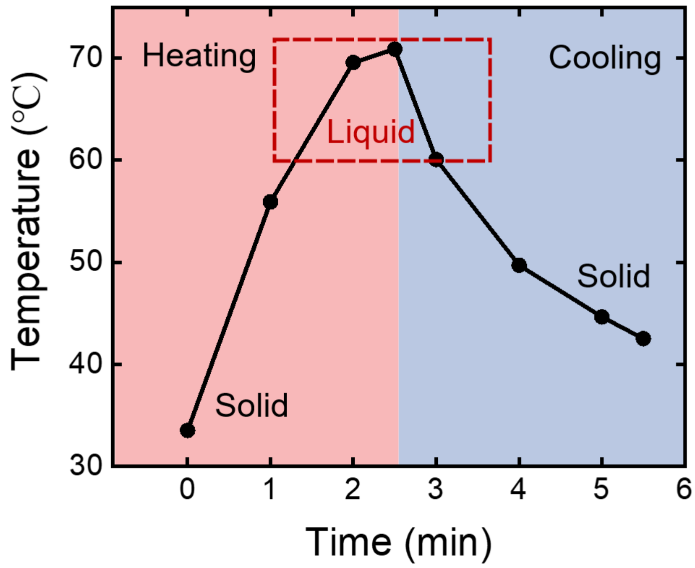

Figure S3. The evolution of temperature during the Joule heating and cooling of the variable stiffness fiber. The curve enclosed within the red box represents the variable stiffness fiber in the liquid state.

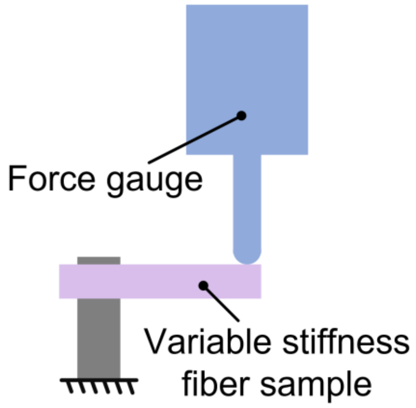

Figure S4. The experimental setup for cantilever beam test.

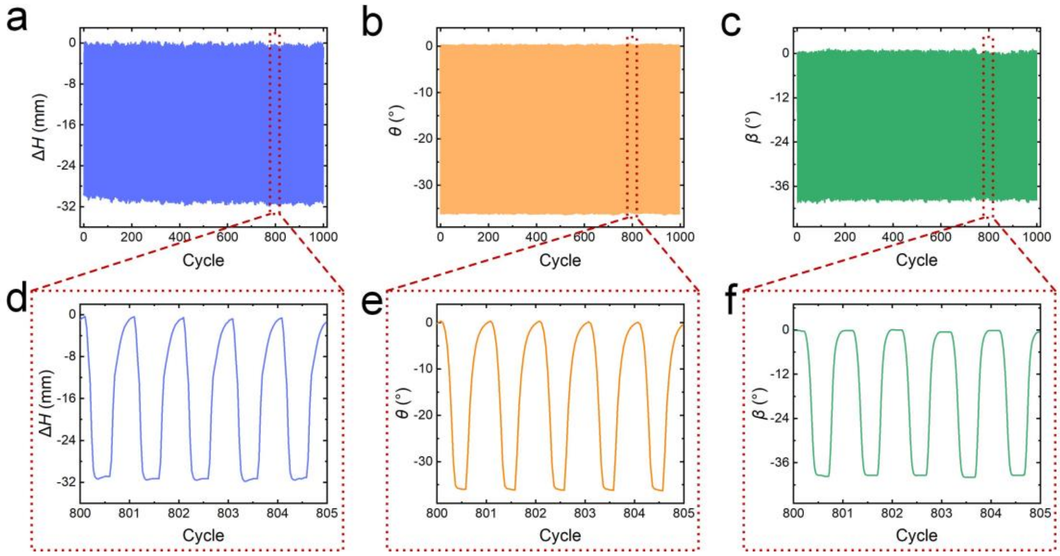

Figure S5. The cyclic testing of the soft origami actuators. (a-c) The continuous operation of the soft circular origami actuator (a), soft rectangular frame actuator (b), and soft square-twist actuator (c) in 1000 cycles. (d-f) The five actuation cycles of the soft circular origami actuator (d), soft rectangular frame actuator (e), and soft square-twist actuator (f) after 800 cycles.

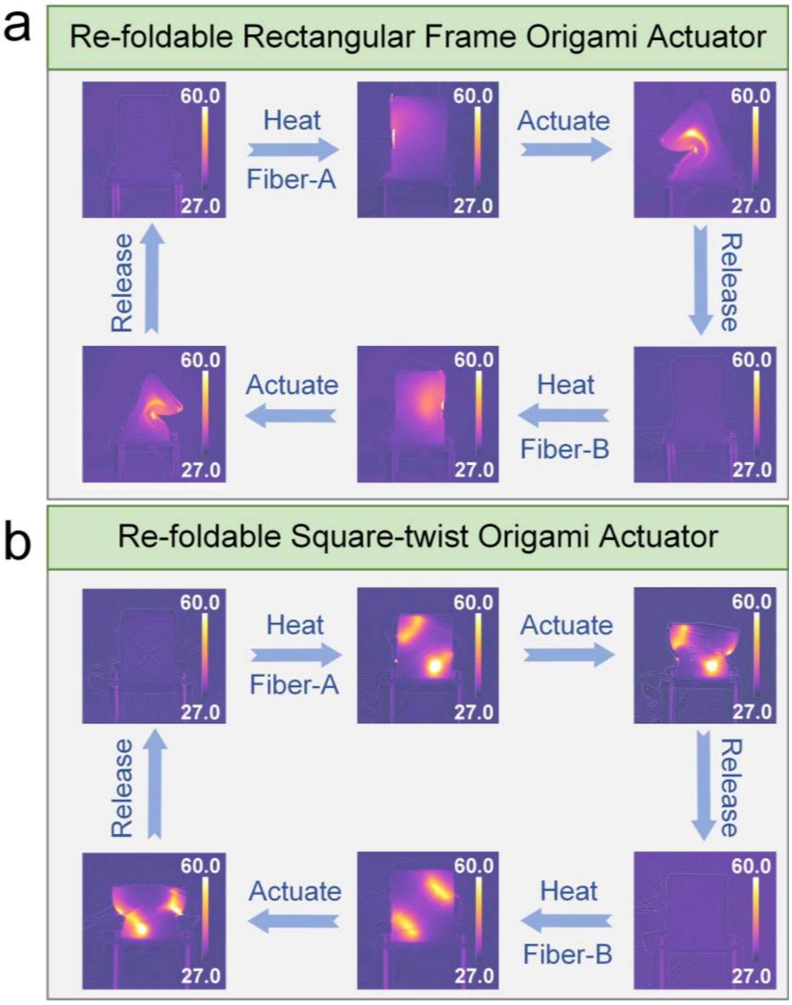

Figure S6. The infrared thermal images of the re-folding process of the soft origami actuators. (a) The rectangular frame origami actuator. (b) The square-twist origami actuator.

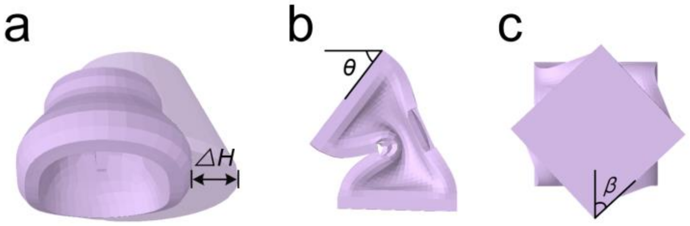

Figure S7. The deformation diagram of the soft origami actuations. (a) The contraction stroke $\triangle H$ of the soft circular origami actuator. (b) The bending angle θ of the soft rectangular frame origami actuator. (c) The twisting angle β of the soft square-twist origami actuator.

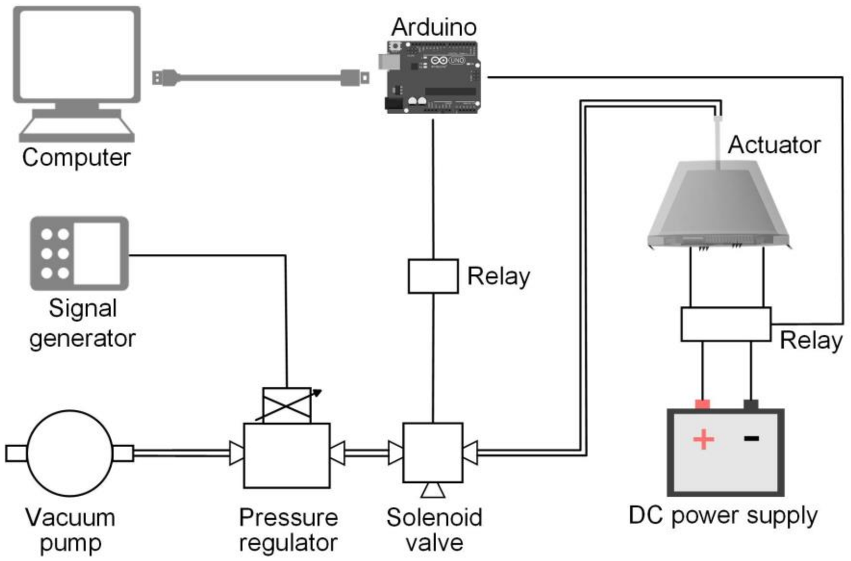

Figure S8. The control system for the re-foldable origami actuators.

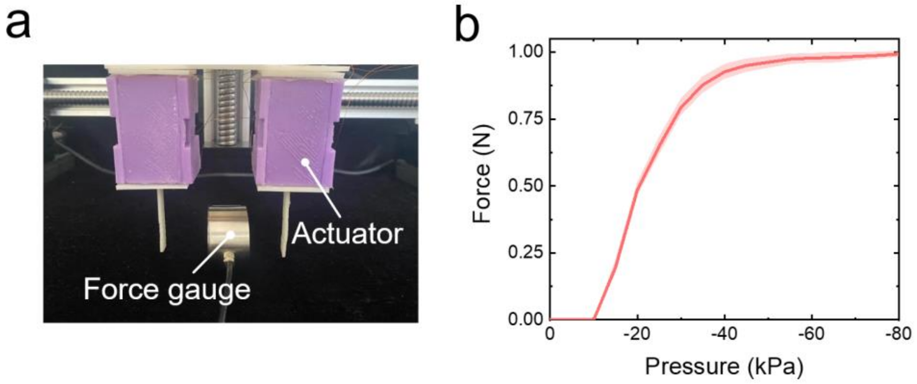

Figure S9. Grasping performances of the soft gripper. (a) The experimental setup for grasping force test. (b) The relationship between grasping force and vacuum pressure demonstrates that the grasping force of the soft gripper can be adjusted.

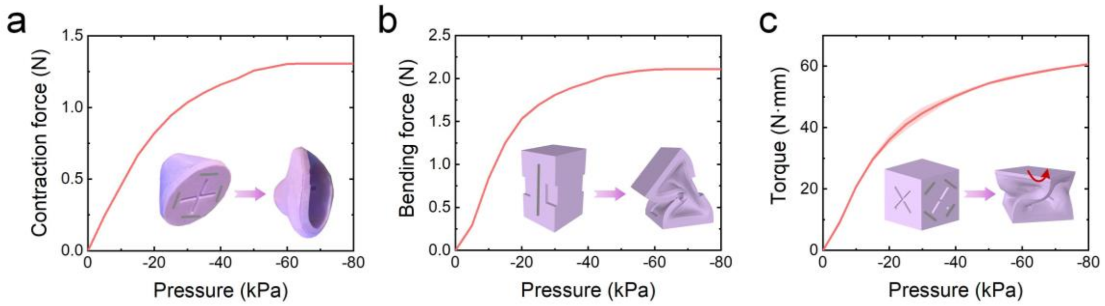

Figure S10. The contraction force, bending force, and torque characterizations of the re-foldable soft origami actuators. (a) The contraction force of the soft circular origami actuator as a function of vacuum pressure. (b) The bending force of the soft rectangular frame origami actuator as a function of vacuum pressure. (c) The torque of the soft square-twist origami actuator as a function of vacuum pressure.

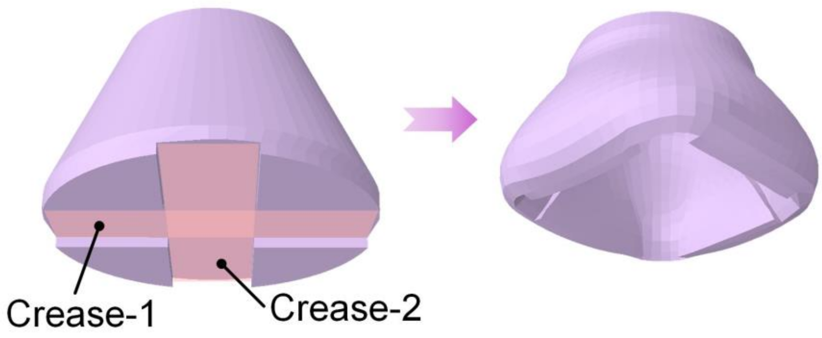

Figure S11. The soft origami actuator with two crossed crease collapses inward when subjected to vacuum pressure.

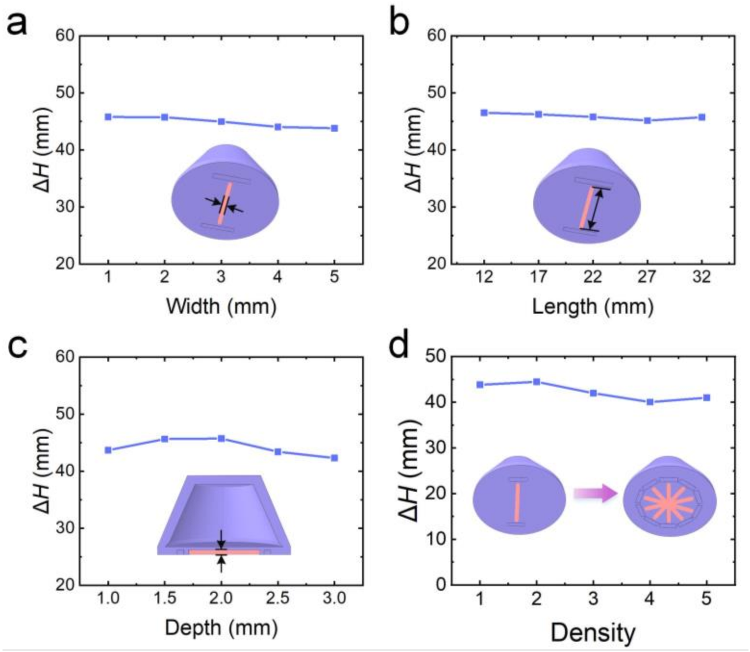

Figure S12. The influence of crease width, length, depth, and density on the maximum deformation of the actuators.

Table S1. The dimensions of the variable stiffness fibers embedded in the actuators.

Actuator	Length (mm)	Width (mm)	Height (mm)
Soft circular origami actuator	20	2	2
Soft rectangular frame origami actuator	50	14	2
Soft square-twist origami actuator	15	2	2

The numerical values used to calculate the melting time are listed as follows:

$$t = \frac{(m_{ela}c_{ela} + m_{LMPA}c_{LMPA})\Delta T + m_{LMPA}L_{LMPA}}{\eta I_{heat}^{2} R - hA\Delta T}$$

The mass of the elastomer: $m_{ela} = 1.72 \times 10^{-3}$ kg

The specific heat of the elastomer: $c_{ela} = 1250$ J/(kg·K)

The mass of the LMPA: $m_{LMPA} = 1.02 \times 10^{-3}$ kg

The specific heat of the LMPA: c_{LMPA} = 172 J/(kg·K)

The latent heat of the LMPA: L_{LMPA} = 39980 J/kg

The heat transfer coefficient of air: h = 10 W/(m^2·K)

The heat transfer surface area: $A = 2.12 \times 10^{-4}$ m^2

The heating efficiency: η = 15%

$$R = \frac{\rho_{wire} l_{wire}}{S} = \frac{\rho_{wire} l_{wire}}{\pi r_{wire}^2}$$

The resistivity of the Ni-Cr resistance wire: $\rho_{wire} = 1.1 \times 10^{-6}$ Ω·m

The length of the Ni-Cr resistance wire: l_{wire} = 0.19 m

The radius of the Ni-Cr resistance wire: $r_{wire} = 5 \times 10^{-5}$ m

Supplementary Movie Captions

Movie S1. The re-foldable circular origami actuator. The variable stiffness fibers enable a soft circular origami actuator to fold along two intersecting creases.

Movie S2. The re-foldable rectangular frame origami actuator. The variable stiffness fibers enable a soft rectangular frame origami actuator to bend bidirectionally.

Movie S3. The re-foldable square-twist origami actuator. The variable stiffness fibers enable a soft square-twist origami actuator to twist bidirectionally.

Movie S4. The re-foldable circular origami actuator with multiple crease patterns. The variable stiffness fibers selectively activate and deactivate four crease patterns, thereby exhibiting more morphing modes.

Movie S5. The re-foldable origami actuator with different types of crease patterns. The bending and twisting crease patterns are deployed into one soft origami actuator to perform rightward bending, leftward bending, anticlockwise twisting, and clockwise twisting.

Movie S6. The re-foldable rectangular frame origami actuators with different dimensions. The soft origami actuators can be scaled up and scaled down to satisfy

various requirements.

Movie S7. The bidirectional movements of a soft crawling robot. The re-foldable circular origami actuator allows a crawling robot to move forward and left.

Movie S8. The soft gripper with multimode morphing capability. A re-foldable square-twist origami actuator and two re-foldable rectangular frame origami actuators are combined to enable a soft gripper capable of adapting to object size, grasping orientation, and placing orientation.

Movie S9. The soft gripper unscrewed a bottle cap. The gripper exhibited continuous rotation by repeatedly executing four consecutive operations: grasping the cap, twisting anticlockwise, releasing the cap, and restoring the soft joint.
